# A Type I Interferon and IL-10 Induced by *Orientia tsutsugamushi* Infection Suppresses Antigen-Specific T Cells and Their Memory Responses

**DOI:** 10.3389/fimmu.2018.02022

**Published:** 2018-09-04

**Authors:** Chan-Ki Min, Hong-II Kim, Na-Young Ha, Yuri Kim, Eun-Kyung Kwon, Nguyen Thi Hai Yen, Je-In Youn, Yoon Kyung Jeon, Kyung-Soo Inn, Myung-Sik Choi, Nam-Hyuk Cho

**Affiliations:** ^1^Department of Microbiology and Immunology,Seoul National University College of Medicine, Seoul, South Korea; ^2^Department of Biomedical Sciences,Seoul National University College of Medicine, Seoul, South Korea; ^3^Wide River Institute of Immunology, Seoul National University College of Medicine, Gangwon-do, South Korea; ^4^Department of Pathology, Seoul National University College of Medicine, Seoul, South Korea; ^5^Department of Pharmaceutical Science, College of Pharmacy, Kyung Hee University, Seoul, South Korea; ^6^Institute of Endemic Disease, Seoul National University Medical Research Center and Bundang Hospital, Seoul, South Korea

**Keywords:** scrub typhus, *Orientia tsutsugamushi*, type I interferon, IL-10, T cells, memory response, cell-mediated immunity

## Abstract

Despite the various roles of type I interferon (type I IFN) responses during bacterial infection, its specific effects *in vivo* have been poorly characterized in scrub typhus caused by *Orientia tsutsugamushi* infection. Here, we show that type I IFNs are primarily induced via intracellular nucleic acids sensors, including RIG-I/MAVS and cGAS/STING pathways, during *O. tsutsugamushi* invasion. However, type I IFN signaling did not significantly affect pathogenesis, mortality, or bacterial burden during primary infection *in vivo*, when assessed in a mice model lacking a receptor for type I IFNs (IFNAR KO). Rather, it significantly impaired the induction of antigen-specific T cells and reduced memory T cell responses. IFNAR KO mice that recovered from primary infection showed stronger antigen-specific T cell responses, especially Th1, and more efficiently controlled bacteremia during secondary infection than wild type mice. Enhanced IL-10 expression by macrophages in the presence of type I IFN signaling might play a significant role in the suppression of antigen-specific T cell responses as neutralization or knock-out (KO) of IL-10 increased T cell responses *in vitro*. Therefore, induction of the type I IFN/IL-10 axis by *O. tsutsugamushi* infection might play a significant role in the suppression of T cell responses and contribute to the short longevity of cell-mediated immunity, often observed in scrub typhus patients.

## Introduction

Type I interferons (type I IFNs), including IFN-α and IFN-β, have diverse effects on innate and adaptive immune responses during viral and bacterial infections (1–3). Even though the antiviral role of type I IFNs has been well-established (1), they are now known to have a myriad of effects in infectious diseases and other immuno-pathological conditions, directly and/or indirectly through the induction of inflammatory mediators (2, 4, 5). In particular, the effect of type I IFN signaling induced during bacterial infection is associated with various downstream beneficial or detrimental consequences for the host depending on the type of bacterial pathogen (3, 6). Therefore, it is critical to distinguish the specific pathogen type- and context-dependent effects of the type I IFN responses to understand the underlying mechanisms of immune modulation during bacterial infection, as well as to design preventive and therapeutic measures for each specific infectious disease.

Expression of type I IFNs by bacterial infection can be initiated by recognition of pathogen-associated molecular patterns (PAMPs), such as nucleic acids, cell wall components, and lipoproteins derived from invading pathogens. These bacterial PAMPs induce type I IFN responses through multiple signaling pathways of various pattern recognition receptor (PRR) systems, including Toll-like receptors (TLRs)-myeloid differentiation factor 88 (MyD88)/TIR domain-containing adaptor-inducing IFN-β (TRIF), retinoic acid-inducing gene-I (RIG-I)-mitochondrial antiviral-signaling protein (MAVS), and the cyclic GMP-AMP synthase (cGAS)-stimulator of IFN genes (STING) pathways (3, 7). All these pathways eventually turn on type I IFNs gene expression, as well as other pro-inflammatory cytokines, via activation of transcription factors, IFN-regulatory factors (IRFs) and nuclear factor-κB (NF-κB) (2). Secreted type I IFNs bind to, and signal through a heterodimeric receptor, composed of IFNAR1 and IFNAR2 (2). Downstream signaling of IFNAR1 and IFNAR2 activates transcription factors, STAT1, STAT2, and IRF9, to form the IFN-stimulated gene factor 3 (ISGF3) complex, which binds to IFN-stimulated response elements (ISREs) in gene promoters, leading to induction of a large number of IFN-stimulated genes (ISGs) (2). Depending on the type of stimulus, the strength and durability of type I IFN production may vary and positively or negatively influence innate immune cell activation and regulation of adaptive immune responses. The reasons and molecular details for the dual actions of type I IFNs in bacterial infections remain poorly understood (8).

Scrub typhus is an acute febrile illness caused by infection with *Orientia tsutsugamushi* (9). This bacterium is an obligate intracellular pathogen transmitted from infected chigger mites to humans (10). The disease is currently a growing threat in Asia and the western Pacific region due to its rising incidence and continuous local outbreaks. In addition, scrub typhus is also emerging in unexpected geographical regions such as South America and Africa (11, 12), where disease endemicity has not been previously reported. Even though early diagnosis followed by appropriate antibiotic therapy can efficiently control the febrile illness, several problems, including relatively high mortality in untreated patients or after delayed diagnosis (13), potential antibiotic resistance (14), recurrent infection in highly endemic areas (15), and growing urbanization primarily due to ecological changes of mite vectors (16), pose challenges in the endemic area. Moreover, an effective vaccine for human infection is not yet available despite continuous efforts since the 1940s (17, 18).

*O. tsutsugamushi* infects human when chiggers feed on tissue fluid and disseminates systemically, targeting multiple organs such as the lung, kidney, liver, brain, and spleen (19). The intracellular pathogen has tropism for dendritic cells, monocytes/macrophages, and endothelial cells (10), where it replicates in the cytosol and induces multiple inflammatory mediators. Additionally, systemic *O. tsutsugamushi* infection in humans causes neutrophilia and CD4 T lymphopenia in the acute phase, followed by proliferation of CD8 T cells with activated phenotype during convalescent phase (20). Such potent immuno-pathological changes in innate and adaptive immune system might be associated with clinical presentations of scrub typhus such as eschar, fever, rash, lymphadenopathy, systemic vasculitis, and multi-organ failure often observed in fatal cases. It is also notable that adaptive immunity generated by primary infection generally rapidly wanes and does not last longer than a few years after infection (21). Particularly, cellular immunity, including CD4 and CD8 T cells specific to *O. tsutsugamushi* antigens, quickly decline from 1 year after infection (21). The short longevity of antigen-specific adaptive immunity might be attributable to limited memory responses, as observed in early vaccine studies using whole bacterial antigens as well as in human patients. Nevertheless, the underlying mechanisms of the short immune memory are poorly understood and remain to be elucidated for developing protective and long-lasting immunity.

Several studies have reported that *O. tsutsugamushi* induces type I IFN responses in monocytes/macrophages and dendritic cells *in vitro* (22, 23), as well as in peripheral blood mononuclear cells from scrub typhus patients (24). In addition, expression of type I IFNs induced by *O. tsutsugamushi* infection in dendritic cells is significantly higher than those by other intracellular bacteria, including *Coxiella burnetii* and *Brucella abortus*, both of which reside in vacuolar compartments (23). Since the effect of type I IFNs on the intracellular replication of *O. tsutsugamushi* is generally marginal (25), further studies are required to determine the exact role of type I IFNs in *O. tsutsugamushi* infection. Here, we investigate the signaling pathways involved in induction of type I IFNs by *O. tsutsugamushi* using several genetic knock-out (KO) systems and search for the potential effects of type I IFN signaling on bacterial pathogenesis as well as on antigen-specific adaptive immunity using mutant mice lacking a receptor subunit for type I IFNs, IFNAR1.

## Materials and methods

### Ethics statement

Animal experiments were approved by the Seoul National University Institutional Animal Care and Use Committee (SNU IACUC, Permit No. SNU-100414-1) and performed in strict accordance with the recommendations in the National Guide Line for the care and use of laboratory animals.

### Mice

Type I IFN receptor α-chain knock-out (IFNAR KO) 129/SvEv mice (26) were kindly provided by Dr. Heung Kyu Lee (Korea Advanced Institute of Science and Technology) and backcrossed with C57BL/6J more than seven generations. Splenocytes and bone marrow cells from MyD88-, TRIF- [MyD88 KO (27), TRIF KO (28)], and IL-10-deficient C57BL/6 mice were provided by Dr. Jong-Hwan Park in Chonnam National University. MAVS knock-out [MAVS KO (29)] mice on C57BL/6 background were generously provided by Dr. Shizuo Akira (Osaka University). IFNAR KO and wild type C57BL/6 mice (Orient Bio, Seongnam, South Korea) were housed and maintained in the specific pathogen-free facility at Seoul National University College of Medicine.

### Cell culture

L929 mouse fibroblast cells were obtained from American Type Culture Collection (Rockville, MD, USA) and cultured in complete Dulbecco's modified Eagle's medium (DMEM, Gibco, Grand Island, NY, USA) containing 10% (vol/vol) heat-inactivated fetal bovine serum (FBS, Gibco), 100 μg/ml of streptomycin, 100 U/ml of penicillin in humidified 5% CO_2_ atmosphere at 37°C. Mouse embryonic fibroblasts (MEF) were isolated from wild type and various KO mice. Embryos were isolated at E13.5 and were chopped and treated with 0.5% Trypsine-EDTA for 5 min at 37°C. Isolated MEF cells were cultured in humidified 5% CO_2_ atmosphere at 37°C. MEF cells derived from RIG-I or STING-deficient mice were kindly provided by Dr. Jae U. Jung (University of Southern California). Bone marrow-derived macrophages (BMDMs) were generated from the bone marrow of 6- to 12-week-old wild type or various KO C57BL/6 mice as previously described (30). Briefly, bone marrow cells were flushed out of femurs and tibias with serum-free DMEM, filtered through a nylon cell strainer (70-μm Nylon mesh; BD Biosciences, San Jose, CA, USA), and washed twice with serum-free DMEM. The cells were then cultured for 4 days in complete DMEM containing 10% L929 cell culture media as a source of M-CSF. Bone marrow-derived dendritic cells were also generated from the bone marrow cells by culturing them with complete Iscove's Modified Dulbecco's Medium (IMDM, Gibco) supplemented with 10% FBS, recombinant mouse GM-CSF (1.5 ng/ml; PeproTech, Rocky Hill, NJ, USA) and mouse IL-4 (1.5 ng/ml; PeproTech), penicillin (100 units/ml), streptomycin (100 μg/ml), gentamicin (50 μg/ml), L-glutamine (2 mM), and β-mercaptoethanol (50 nM; Gibco Invitrogen) for 6 days (31). Differentiated BMDCs were harvested using the CD11c microbeads kit (MACS, Gladbach, Germany).

### Preparation of *orientia tsutsugamushi* and infection study

*O. tsutsugamushi* Boryong strain was purified using a modified Percoll gradient purification method (17). *O. tsutsugamushi* was propagated in L929 cells. At 3–4 days post-infection, infectivity was determined using an indirect immunofluorescence assay. When an infection rate of >90% was achieved, the cells were harvested by centrifugation at 500 × g for 4 min. The cell pellet was resuspended with 6.5 ml of Tris-sucrose (TS) buffer (33 mM Tris-Cl [pH 7.4], 0.25 M sucrose) and the cells were homogenized using 100 strokes of a Polytron homogenizer (Wheaton Inc., Millville, NJ, USA) followed by centrifugation at 200 × g for 5 min. The supernatant was then mixed with 40% Percoll (Pharmacia Fine Chemicals, Uppsala, Sweden) in TS buffer and centrifuged at 25,000 × g for 60 min. The bacterial band was collected and centrifuged at 77,000 × g for 30 min. The bacterial pellet was washed three times in TS buffer, resuspended in DMEM and stored in liquid nitrogen until use. The infectivity titer of the inoculum was determined as previously described (17). For infection assays, cell cultures in 24 well plate were infected with 2.5 × 10^6^ infected-cell counting unit (ICU) (17) of *O. tsutsugamushi* (~4 bacteria/cell). In every infection study, we confirmed that more than 90% of the cells were infected with *O. tsutsugamushi* after 2 h of incubation. Fifty percent of lethal dose (LD_50_) were determined in wild type C57BL/6J.

### Histopathologic analysis of infected tissues

All the isolated tissues were fixed in 4% paraformaldhehyde (Sigma-Aldrich, St. Louis, MO, USA) and embedded in paraffin. Tissue sections (10 μm thickness) were stained with hematoxylin and eosin. Stained lung tissue sections were scanned and scored at the pathology core facility of Seoul National University College of Medicine, following standard procedures. We used a 0–4 scoring system: grade 0, normal; grade 1, widening of alveolar septa with scattered inflammatory cells in focal areas of pulmonary parenchyma and focal inflammatory cells around bronchovascular bundles (<10% of lung); grade 2, widening of alveolar septa with scattered inflammatory cells in multifocal areas of pulmonary parenchyma and around bronchovascular bundles (10–50% of lung); grade 3, widening of alveolar septa with diffuse inflammatory cell infiltrates present in the pulmonary parenchyma and bronchovascular bundles (more than 50% of lung); grade 4, grade 3 criteria plus area of atelectasis.

### Determination of bacterial load

Bacterial loads of infected tissues were assessed by quantitative real-time PCR (qRT-PCR) as previously described (32). Briefly, DNA was extracted from the tissue samples using a DNeasy Kit (Qiagen, Gaithersburg, MD, USA), and the bacterial load was determined by using a primer set derived from the 47 kDa gene: p47 forward (5′-AACTGATTTTATTCAAACTAATGCTGCT-3′), p47 reverse (5′-TATGCCTGAGTAAGATACATGAATGGAATT-3′), and detecting probe (5′-6FAM-TGGGTAGCTTTGGTGGACCGATGTTTAATCT-TAMRA-3′). Bacterial loads were normalized to total μg of DNA per ml for the same sample and expressed as the number of 47 kDa gene copies per μg of total DNA.

### RNA purification and quantitative reverse transcriptase PCR (qRT-PCR)

Total RNA was extracted from cells using Trizol reagent (Sigma-Aldrich, St. Louis, MO, USA) according to the manufacturer's instruction. Approximately 1 microgram of total RNA was reverse transcribed by Eco-dry cDNA Synthesis kit containing poly-dT primer (Clonetech, Mountain View, CA, USA). The quantification of cDNA was performed with gene specific primers using Power SYBR green PCR Master Mix (Applied Biosystems, Grand Island, NY, USA) and processed using the ABI 7500 (Applied Biosystems). The primer sequences are as follows: β-actin (forward: GTGACGTTGACATCCGTAAAGA, reverse: GCCGGACTCATCGTACTCC), IFN-β3 (forward: ATGGTGGTCCGAGCAGAGAT, reverse: CCACCACTCATTCTGAGGCA), and TNF-α3 (forward: TCCCCAAAGGGATGAGAAGTT, reverse: GTTTGCTACGACGTGGGCTAC). The relative level of gene expression was calculated by the 2^−dCt^ or the ddCt method (33), where β-actin transcripts was used for normalization. The qRT-PCR data represent the average of three independent experiments.

### Cytokine assay

The concentrations of cytokines in sera from infected mice or cell cultures were measured using mouse cytokine/chemokine magnetic bead panel 96-well plate assay according to the manufacturer's instructions (Merck Millipore, Darmstadt, Germany). The cytokines analyzed in this study are tumor necrosis factor (TNF-α), interferon-γ (IFN-γ), interleukin-2 (IL-2), IL-4, IL-6, IL-10, IL-1β, and IL-12(p70). Concentration of secreted IFN-β in cell culture supernatants were determined by ELISA (R&D systems, Minneapolis, MN, USA) according to the manufacturer's instruction.

### Enzyme-linked immunosorbent assay (ELISA)

The level of antibodies specific to TSA56 in the sera of infected mice was analyzed ELISA as previously described (34). Immunoassay plates (96-well plates; Nunc, Rochester, NY, USA) were coated with 100 μl of purified antigen at a concentration of 5 μg/ml at 4°C overnight. The plates were then blocked for 2 h at room temperature with PBS containing 1% BSA. Hundred microliters of serum samples serially diluted in two-fold were incubated for 2 h at room temperature. After washing with PBS containing 0.05% Tween20 (PBST), horseradish peroxidase (HRP)-conjugated goat anti-mouse IgG (Santa Cruz Biotechnology, Santa Cruz, CA, USA) was added and incubated for 2 h at room temperature. Wells were washed with PBST and incubated with 3,3′,5,5′-tetramethylbenzidine (TMB) peroxidase substrate solution (KPL, Gaithersburg, MD, USA) for 10 min. The reactions were stopped by addition of 1 M phosphoric acid solution. Absorbance was measured at 450 nm using a microplate reader (Beckman Coulter Inc., Fullerton, CA, USA).

### Type I IFN bioassay

Cell-culture supernatants from stimulated cells or sera from infected mice were incubated with L929 cells containing a stable IFN-stimulated response element-luciferase reporter plasmid [ISRE-luc (35)] for 4 h. The reporter cells were lysed in Passive Lysis Buffer (Promega, Madison, WI, USA) for 30 min at room temperature, mixed with firefly luciferin substrate (Promega), and measured on a luminometer (Becman coulter, Fullerton, CA, USA).

### Cytokine neutralization assay

Splenocytes (2 × 10^6^ cells/24-well) isolated from IFNAR KO mice were infected with *O. tsutsugamushi* for 1 day and further incubated in the presence of tetracycline (0.3 μg/ml) for 3 days. Neutralizing monoclonal antibodies, such as anti-IL-1β (clone B122, eBioscience, San Diego, CA, USA), anti-IL-6 (clone MP5-20F3, eBioscience), and anti-IL-10 (clone JES5-2A5, Biolegend, San Diego, CA, USA), as well as isotype control antibody (murine IgG_1_, Biolegend), were added to the culture media (10 μg/ml each/24-well) of the infection assays. Cells were then stimulated with 10 μg of TSA56 for an additional 18 h and 1 μg of Golgiplug (BD Bioscience) for the final 6 h in humidified CO_2_ atmosphere at 37°C. Harvested splenocytes were stained with specific antibodies and analyzed by flow cytometry as described below.

### Flow cytometry

Spelnocytes were stained with antibodies against the indicated surface molecules after blocking on ice for 30 min with ultra-block solution containing 10% rat sera, 10% hamster sera, 10% mouse sera (Sigma, St. Louis, MO, USA), and 10 μg/ml of anti-CD16/32 (2.4G2; BD Pharmingen, Franklin Lakes, NJ, USA). Anti-CD44 (IM7, from Biolegend), CD3 (145-2CD11), CD4 (RM4-5), CD69 (H1.2F3), CD8 (53-6.7) (from eBioscience), CD62L (MEL-1, from BD Pharmingen) antibodies, and annexin V (BD Pharmingen) conjugated to differential fluorescent dyes were used for flow cytometric analysis. Cells were also stained with 7-AAD (BD Pharmingen) for dead cell exclusion in some experiments. For intracellular detection of IFN-γ and TNF-α, splenocytes (1 × 10^6^ cells) were stimulated with 10 μg of purified TSA56 antigen and 1 μg Golgiplug (BD Bioscience) for the final 6 h in humidified CO_2_ atmosphere at 37°C. Stimulated cells were then stained with the indicated surface markers. Surface-stained cells were fixed and permeabilized with Fixation and Permeabilization Solution (BD Bioscience), followed by incubation with anti-IFN-γ (XMG1.2; BD Pharmingen) and TNF-α (MF6-XT22, Affymetrix, Cleveland, OH) antibodies. Fluorescence intensities of the stained molecules were examined on a FACS Fortessa II flow cytometer (BD Biosciences). Data were analyzed using Flowjo software (Tree Star, Ashland, OR, USA). Gating strategies for the flow cytometric analysis are summarized in Figure S1 (Supplementary data).

### Statistical analysis

The data was analyzed using Graph Pad Prism 5.01 software (GraphPad Software, La Jolla, CA, USA). Statistical analysis was performed using two-tailed Student's *t*-test with 95% confidence interval or one-way analysis of variance (ANOVA) followed by Newman-Keuls *t*-test for comparisons of values among different groups. Data are expressed as the mean ± standard deviation. Statistical analysis on survival rates were performed using the Mantel-Cox Log Rank test. A *p*-value of < 0.05 was considered statistically significant.

## Results

### Induction of type I IFN responses by *O. tsutsugamushi* infection

**Figure 1:**
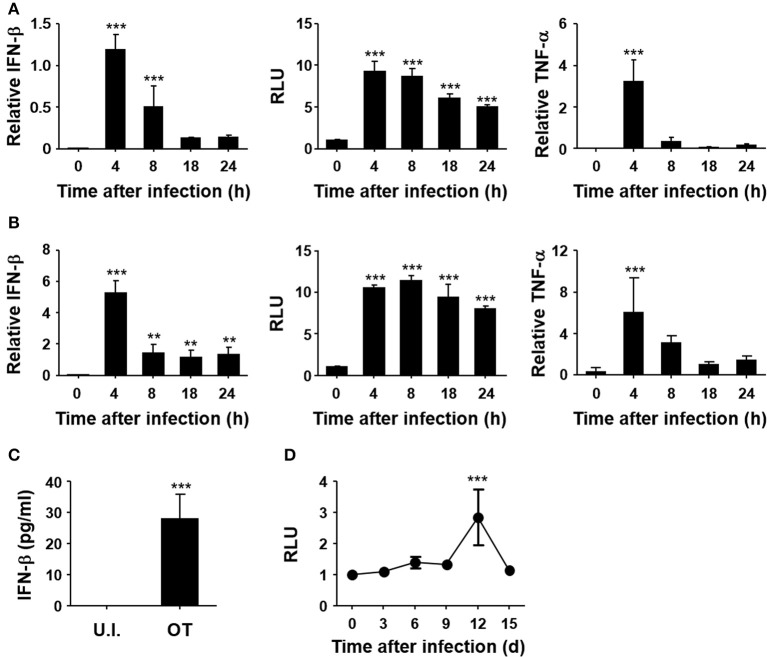
Induction of type I IFN by *O. tsutsugamushi* infection. Mouse embryonic fibroblasts (MEFs, **A**) and bone marrow-derived macrophages (BMDMs, **B**) were infected with *O. tsutsugamushi* and harvested at the indicated time points. Total RNA was isolated from the infected cells and the relative levels of IFN-β (left) and TNF-α (right) transcripts, normalized with β-actin mRNA, were determined by qRT-PCR. Type I IFN bioactivity (middle) was also analyzed in L929 cells containing a stable ISRE-luciferase reporter plasmid after stimulation with culture supernatants collected at the indicated times. **(C)** Secreted IFN-β from infected BMDMs was analyzed by ELISA after 18 h of infection. U.I., uninfected; OT, *O. tsutsugamushi*-infected. **(D)** Mice were infected with 5 × LD_50_ of *O. tsutsugamushi* and type I IFN bioactivity in plasma collected at the indicated times was determined in L929 cells containing a stable ISRE-luciferase reporter plasmid. Data represent mean ± SD of three independent experiments. Statistical significance was determined by one-way analysis of variance (ANOVA) followed by Newman-Keuls *t*-test for comparisons with uninfected control. ^***^p < 0.001, ^**^p < 0.01. RLU, relative luciferase unit.

To confirm the induction of type I IFNs by *O. tsutsugamushi*, non-phagocytic MEFs and phagocytic BMDMs were infected *in vitro* with *O. tsutsugamushi* and the relative levels of IFN-β transcripts were assessed by quantitative real-time PCR. The results demonstrated a rapid upregulation of mRNA expression of IFN-β, as well as TNF-α, in MEFs and BMDMs with peak responses at 4 h after infection (Figures 1A,B). Secretion of type I IFNs from the infected cells was further confirmed by type I IFN bioassay using L929 cells harboring ISRE-luc after stimulation with culture supernatants collected from infected cells at the indicated times (Figures 1A,B, middle). Secreted IFN-β from infected BMDMs was also detectable by ELISA at 18 h after infection (Figure 1C). In addition, we also observed a gradual increase of type I IFN activity in the sera of infected mice, as measured type I IFN bioassay (Figure 1D). These results clearly demonstrate that type I IFN responses are significantly upregulated during *O. tsutsugamushi* infection *in vitro* and *in vivo*.

### Role of intracellular nuclear acid sensor pathways in the induction of type I IFN responses by *O. tsutsugamushi* infection

**Figure 2:**
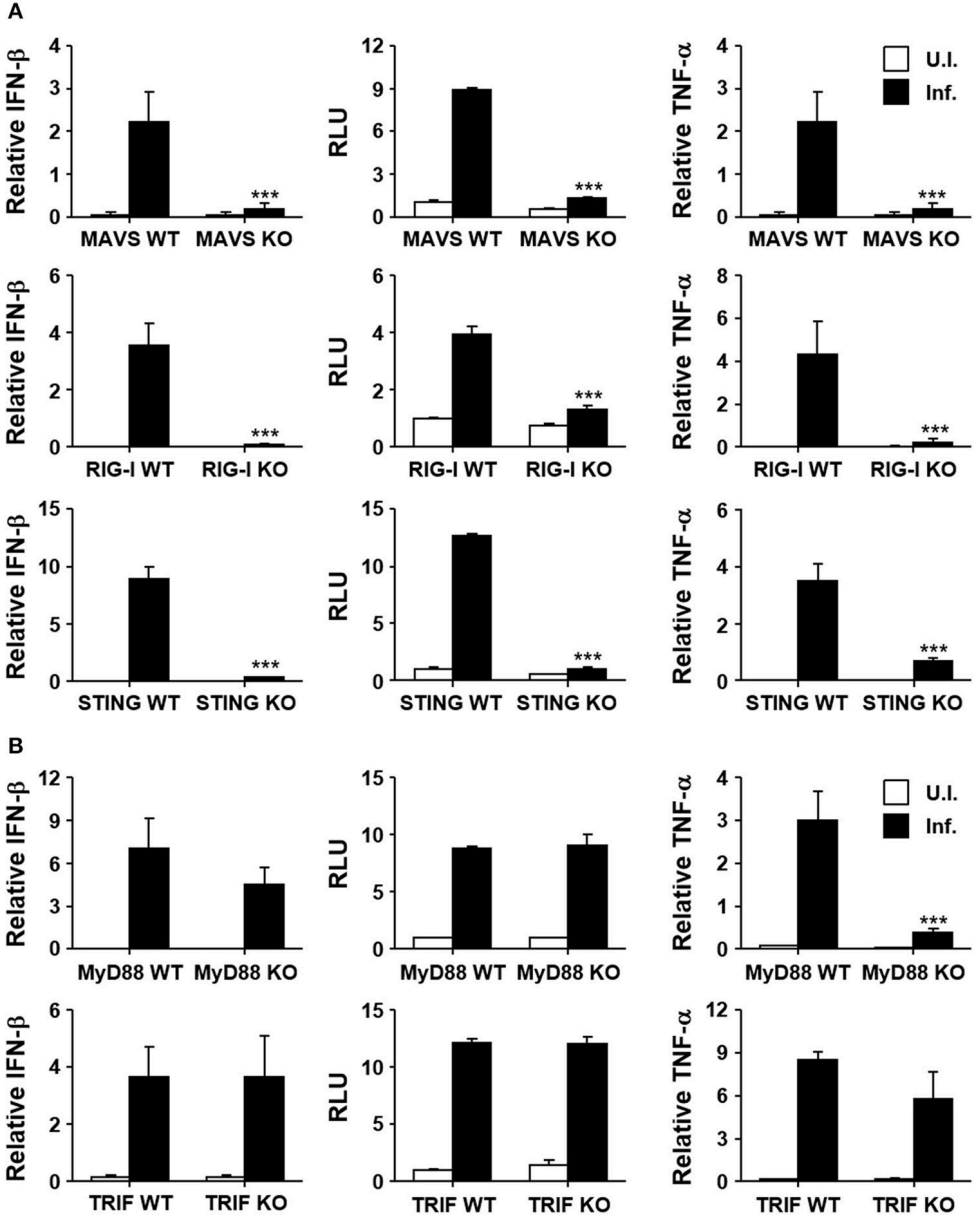
Induction of type I IFN by *O. tsutsugamushi* infection through RIG-I/MAVS and STING. **(A)** MEFs derived from wild type (WT), MAVS-deficient (MAVS KO), RIG-I-deficient (RIG-I KO), or STING-deficient (STING KO) mice were infected with *O. tsutsugamushi* and the relative expression of IFN-β and TNF-α mRNA, as well as type I IFN bioactivity, were analyzed at 4 h after infection as describe in Figure 1. **(B)** BMDMs derived from wild type (WT), MyD88-deficient (MyD88 KO), or TRIF-deficient (TRIF KO) mice were infected with *O. tsutsugamushi* and the relative expression of IFN-β and TNF-α mRNA, as well as type I IFN bioactivity, were determined at 4 h after infection as described in Figure 1. Data represent mean ± SD of three independent experiments. Statistical significance was determined by two-tailed Student's *t*-test with 95% confidence interval for comparisons of values between wild type and KO cells after infection with *O. tsutsugamushi*. ^***^p < 0.001. RLU, relative luciferase unit. White box, uninfected (U.I.); black box, infected (Inf.).

Induction of type I IFNs by the intracellular bacterial pathogen may be mediated by various innate pattern-recognition receptors (PRRs) during the infection process. In order to assess the potential role of diverse PRRs for the induction of type I IFNs in *O. tsutsugamushi* infection, we infected MEFs derived from KO mice lacking MAVS, RIG-I, or STING, and their wild type littermates (Figure 2A). Expression of IFN-β transcripts was severely impaired in MEFs deficient in intracellular nucleic acid sensors and adaptor compared to wild type MEFs. In addition, secretion of type I IFNs after the bacterial infection was abrogated in all the three KO MEFs, as measured by type I IFN bioassays (Figure 2A, middle). It is also notable that expression of TNF-α mRNAs was also drastically suppressed in all the KO MEFs upon bacterial infection, suggesting a significant role of RIG-I/MAVS and STING signaling pathways in non-phagocytic host cells. Since the potential role of other PRRs, including TLRs, for the induction of inflammatory cytokines during the bacterial infection has been reported (36), we examined the expression of type I IFN and TNF-α in professional phagocytic BMDMs lacking MyD88 or TRIF, the essential adaptors for TLR signaling. As seen in Figure 2B, expression of type I IFN was generally intact in BMDMs derived from the two different KO mice and comparable to that of wild type phagocytes. However, expression of TNF-α mRNAs was impaired in MyD88-deficient cells, but not in BMDMs lacking TRIF, indicating a specific role of MyD88 for the induction of TNF-α in professional phagocytes. Taken together, the induction of type I IFN responses during *O. tsutsugamushi* infection might be primarily associated with the RIG-I/MAVS and STING signaling pathways, with minor contribution by TLR signaling mediated by MyD88 and/or TRIF adaptors.

### Role of type I IFN signaling in pathogenesis and bacterial burden during *O. tsutsugamushi* infection *in vivo*

**Figure 3:**
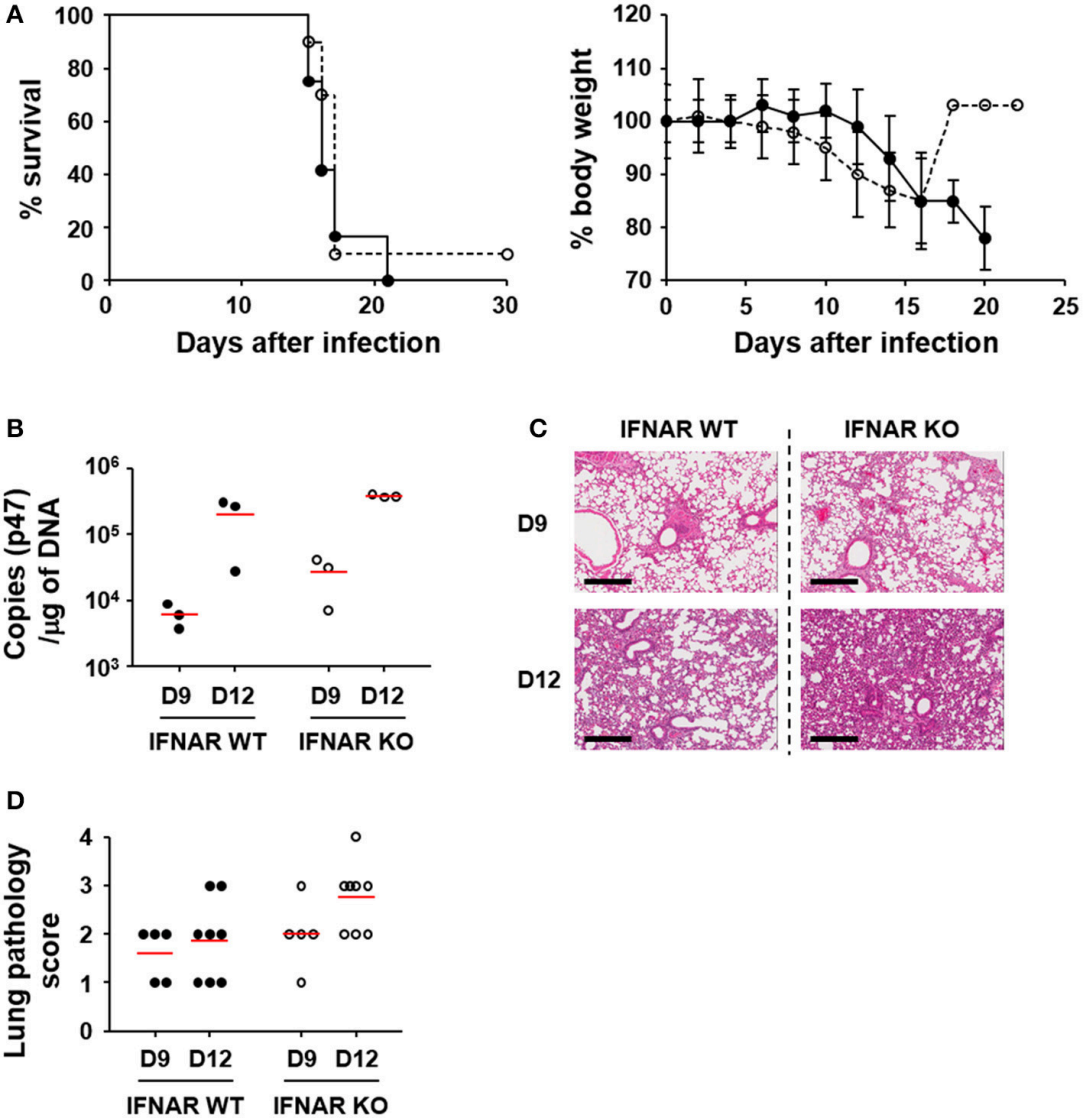
Effect of type I IFN signaling on mortality, pathogenesis, and bacterial loads in *in vivo* infection of *O. tsutsugamushi*. **(A)** Wild type (black circle) and IFNAR-deficient (white circle) mice (*n* = 10) were intraperitoneally infected with 5 × LD_50_ of *O. tsutsugamushi* and survival rate and weight changes (relative to initial body weight on day 0) were monitored for a month. **(B)** Bacterial loads in the lungs of infected mice (wild type or IFNAR KO, *n* = 3/group) were assessed by qRT-PCR using primer sets detecting the p47 gene of *O. tsutsugamushi*. The infected tissues were collected at the indicated days after infection. **(C)** Lung tissue sections collected from mice at the indicated days after infection were stained with hematoxylin and eosin and representative scanned images are presented (see also Figure S2). Bar, 300 μm. **(D)** Pathological scores of infected lungs (D9: *n* = 5, D12: *n* = 8) are presented. Red lines, mean.

In order to assess the effect of type I IFN responses on the pathogenesis of *O. tsutsugamushi* infection, we first evaluated the survival rate of wild type and mutant mice deficient in a receptor subunit for IFN-α and IFN-β (IFNAR KO) after intraperitoneal infection with fatal dose (5 × LD_50_) of *O. tsutsugamushi* (Figure 3A). Both the wild type and IFNAR KO mice similarly succumbed to *O. tsutsugamushi* infection within 3 weeks after infection. The survival rate of the IFNAR KO mice was not significantly different from wild type mice even when infected with lower (1 × LD_50_) or higher (100 × LD_50_) dose of the intracellular pathogen (Figure S2 in Supplementary data). Nevertheless, we consistently observed that IFNAR KO mice more rapidly lost weight than wild type mice, but without statistical significance (Figure 3A, right). Since the lung and spleen are the primary target organs in the mouse infection model for scrub typhus (32), we also measured the bacterial loads and observed histopathologic changes in the infected organs of lethally infected mice at various time points (Figures 3B–D and Figure S3 in Supplementary data). The bacterial burden in the lungs of infected mice gradually increased in both mice groups (Figure 3B). Pathologic examination of the lungs revealed that pulmonary lesions and interstitial pneumonia progressed similarly in wild type and IFNAR KO mice (Figures 3C,D). Even though the bacterial burdens and pathological changes in the lungs of IFNAR KO mice seem to be slightly more severe than wild type mice, those differences were not statistically significant. The spleens were also gradually enlarged with disintegration of follicular structures (Figure S3). The histological changes and the bacterial loads observed in the spleens of infected mice were also not significantly different between wild type and IFNAR KO mice (Figure S3). These results suggest that type I IFN signaling induced by *O. tsutsugamushi* infection does not significantly affect bacterial proliferation *in vivo* and the pathologic process of acute lethal infections.

### Enhanced T cell responses and memory against *O. tsutsugamushi* antigen in the absence of type I IFN signaling

**Figure 4:**
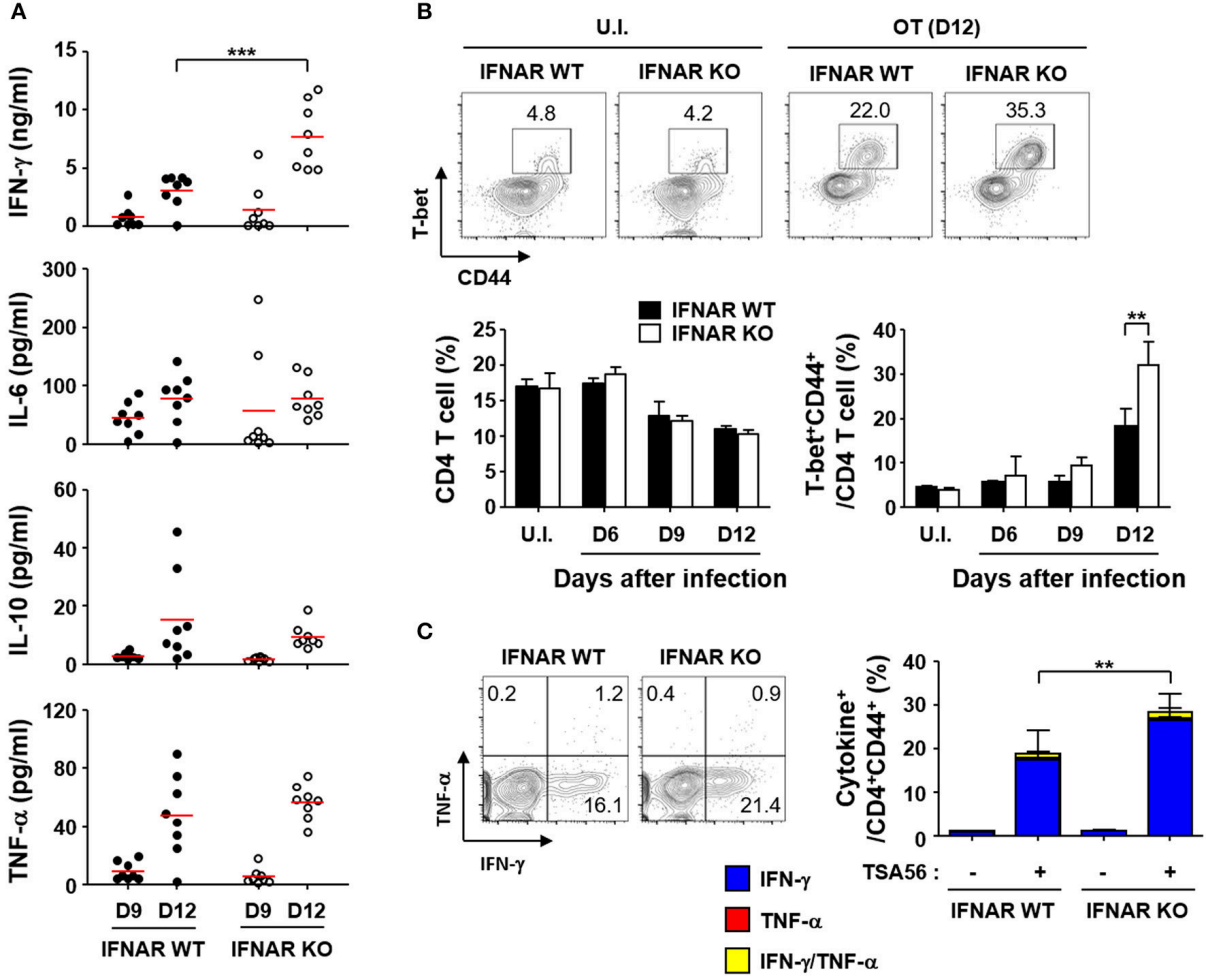
Enhanced IFN-γ, Th1, and CD8 T cell responses by *O. tsutsugamushi* infection in the absence of type I IFN signaling. **(A)** Levels of cytokines in plasma collected at the indicated days after infection in wild type or IFNAR KO mice (*n* = 8/group) were determined. IL-2, IL-4, and IL-12p70 were barely detectable throughout the infection period (data not shown). Red line, mean; ^***^p < 0.001. **(B)** Proportion of T-bet^+^CD44^+^ activated Th1 cells among CD4 T cells were analyzed in spleens of wild type and IFNAR KO mice infected by *O. tsutsugamushi*. Representative images of flow cytometric results are presented (upper) and proportion of CD4 T cells (lower left) and activated Th1 cells (lower right) are summarized (*n* = 3/group). Data represent mean + SD of three independent experiments. ^**^p < 0.01. U.I., uninfected; OT (D12), infected with *O. tsutsugamushi* for 12 days. **(C)** Splenocytes were collected from wild type and IFNAR KO mice at 12 days after infection with *O. tsutsugamushi* and production of IFN-γ and/or TNF-α in activated (CD44^+^) CD4 T cells were analyzed by flow cytometry. Representative flow cytometric results are presented (left) and the percentile of cytokine positive cells among the activated T cell subsets are summarized (right graph) in the absence and presence of TSA56 antigen. Data represent mean + SD from wild type (*n* = 5) or IFNAR KO (*n* = 8) mice. ^**^p < 0.01. Blue box, IFN-γ-positive; red box, TNF-α-positve; yellow box, IFN-γ and TNF-α-positive.

To evaluate whether type I IFN signaling affects immune responses against the bacterial pathogen, we infected wild type and IFNAR KO mice with a lethal dose (5 × LD_50_) of *O. tsutsugamushi* and examined various inflammatory cytokines in the plasma (Figure 4A). The inflammatory cytokines including IFN-γ, IL-6, 1L-10, and TNF-α gradually increased in both mice groups as the disease progressed. Interestingly, the plasma levels of IFN-γ in IFNAR KO mice were significantly higher than those of wild type mice at 12 days after infection, while responses of other inflammatory cytokines were similar between wild type and IFNAR KO mice (Figure 4A). This result prompted us to assess antigen-specific T cell responses in the infected mice since IFN-γ is a hallmark cytokine for Th1 response. We first measured the changes in the fraction of Th1 cells in the infected mice at 12 days after infection. Although the overall fractions of CD4^+^ T cells of both mice groups were similar and significantly reduced by ~35% among spleen lymphocytes at 12 days after infection, T-bet^+^/CD44^+^ activated Th1 cells in the spleens of IFNAR KO mice were significantly higher than those of wild type mice (Figure 4B). Furthermore, the proportion of IFN-γ-secreting Th1 cells was also upregulated among activated (CD44^+^) T cell populations upon stimulation with TSA56, a major outer membrane antigen of *O. tsutsugamushi* (Figure 4C). These results clearly indicate that generation of antigen-specific Th1 lymphocytes were significantly enhanced in the absence of type I IFN signaling during acute bacterial infection.

**Figure 5:**
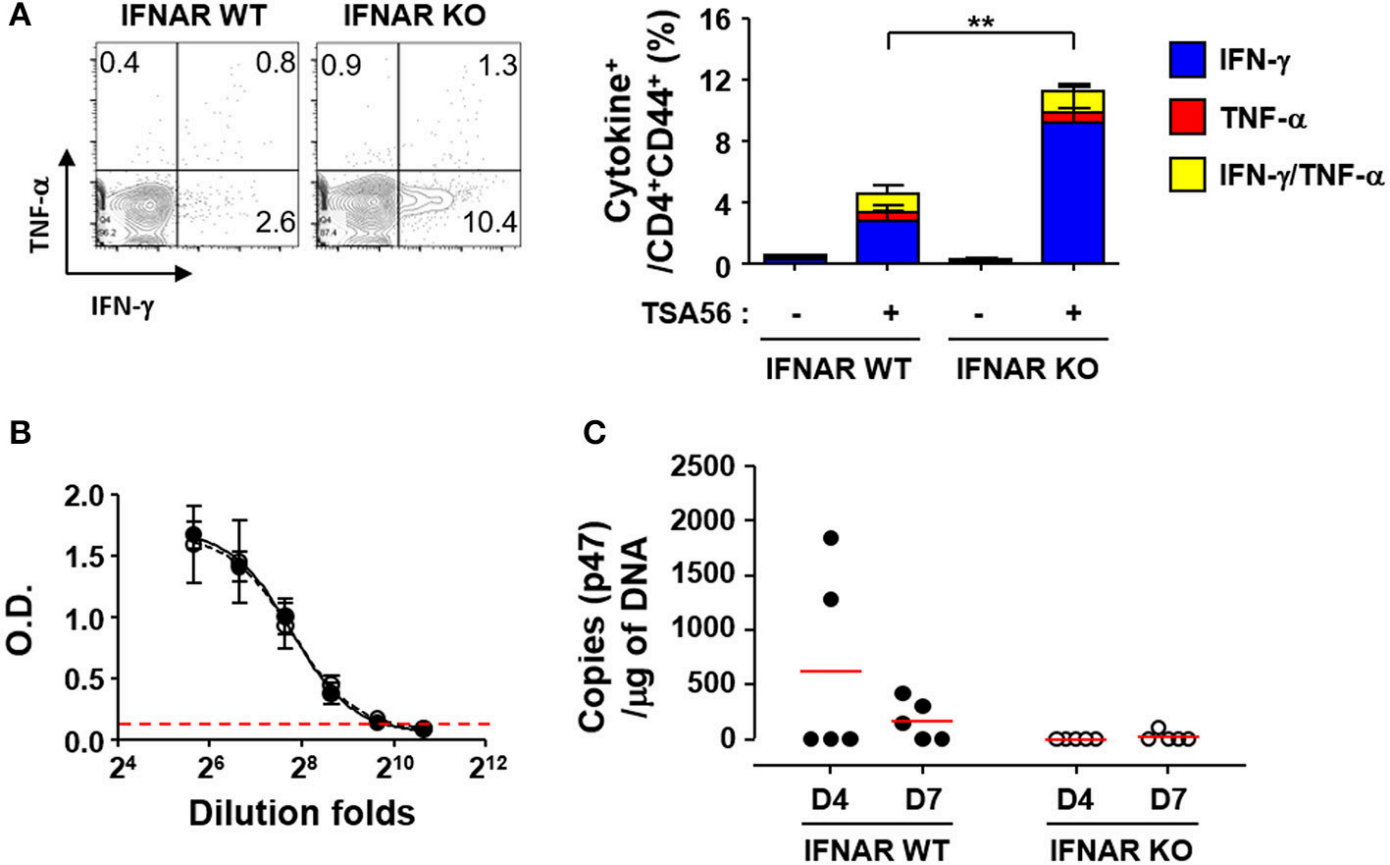
Enhanced T cell memory responses by *O. tsutsugamushi* infection in the absence of type I IFN signaling. **(A)** Splenocytes were collected from cured wild type and IFNAR KO mice at 6 weeks after initial infection with *O. tsutsugamushi* and production of IFN-γ and/or TNF-α in activated (CD44^+^) CD4 T cells were analyzed by flow cytometry. Mice were cured with tetracycline during the second week of lethal infection. Representative flow cytometric results are presented (left) and the percentile of cytokine positive cells among the activated T cell subsets are summarized (right graph) in the absence and presence of TSA56 antigen. Data represent mean + SD from wild type or IFNAR KO (*n* = 5) mice. ^**^p < 0.01. Blue box, IFN-γ-positive; red box, TNF-α-positve; yellow box, IFN-γ and TNF-α-positive. **(B)** Anti-TSA56 antibody (IgG) responses were analyzed in plasma from wild type (closed circles) and IFNAR KO (open circles) mice at 6 weeks after initial infection with *O. tsutsugamushi*. Optical densities (O.D.) of serially diluted samples (*n* = 3/group) were determined by ELISA. Red line: baseline O.D. + 3 × SD of plasma (*n* = 3, 1:100 diluted) from uninfected mice. **(C)** Bacterial loads in the lungs of infected mice (wild type or IFNAR KO, *n* = 5/group) were assessed by qRT-PCR using primer sets detecting the p47 gene of *O. tsutsugamushi*. Mice were again infected with *O. tsutsugamushi* at 6 weeks after the initial infection and the infected lungs were collected at the indicated days after the second infection and analyzed. Red line, mean.

In order to investigate whether enhanced T cell responses in the absence of type I IFN signaling during the acute phase of infection is linked to stronger memory T responses, we challenged the mice with lethal doses (5 × LD_50_) of *O. tsutsugamushi* and treated them with tetracycline at 2 weeks after the initial infection. We confirmed the clearance of the bacterial pathogens by quantitation of bacterial genes in the lungs and spleens of infected mice and by observing mice morbidity during the third week after the pathogen challenge. At 6 weeks after the initial challenge with *O. tsutsugamushi*, splenocytes were collected from the cured mice and assessed for antigen-specific memory T cells by flow cytometry (Figure 5A). We observed there was no significant difference in the relative frequencies and absolute counts of T cells between wild type and IFNAR KO mice (Table S1). However, the proportion of IFN-γ-secreting CD4 T cells (average: 9.2%, *n* = 5) among memory T cells (CD62L^−^/CD44^+^/CD4^+^) in IFNAR KO mice was three times higher than that of wild type mice (average: 2.8%, *n* = 5) when cells were stimulated with TSA56 (Figure 5A). Antibody (IgG) responses specific to TSA56 antigen were similar between wild type and IFNAR KO mice (Figure 5B). These results indicate that type I IFN responses induced by *O. tsutsugamushi* infection specifically suppress antigen-specific Th1 responses, but do not significantly affect humoral immunity against the bacterium. To assess the potential protective role of enhanced cellular memory against *O. tsutsugamushi*, we challenged the cured mice with lethal doses (5 × LD_50_) of *O. tsutsugamushi* at 6 weeks after initial infection and observed the morbidity of infected mice. Both wild type and IFNAR KO mice showed no significant difference in morbidity and weight changes and all the mice were protected from the second challenge. However, *O. tsutsugamushi* was transiently detected in the lungs of several wild type mice at days 4 and 7 after infection, whereas the bacterial gene was barely detected in IFNAR KO mice after the second challenge (Figure 5C). These results suggest that enhanced T cell memory in IFNAR KO mice more efficiently protect them from systemic bacteremia after the second challenge than wild type mice.

### Enhanced expression of IL-10 by type I IFN response is associated with suppression of T cell responses during the acute phase of infection

**Figure 6:**
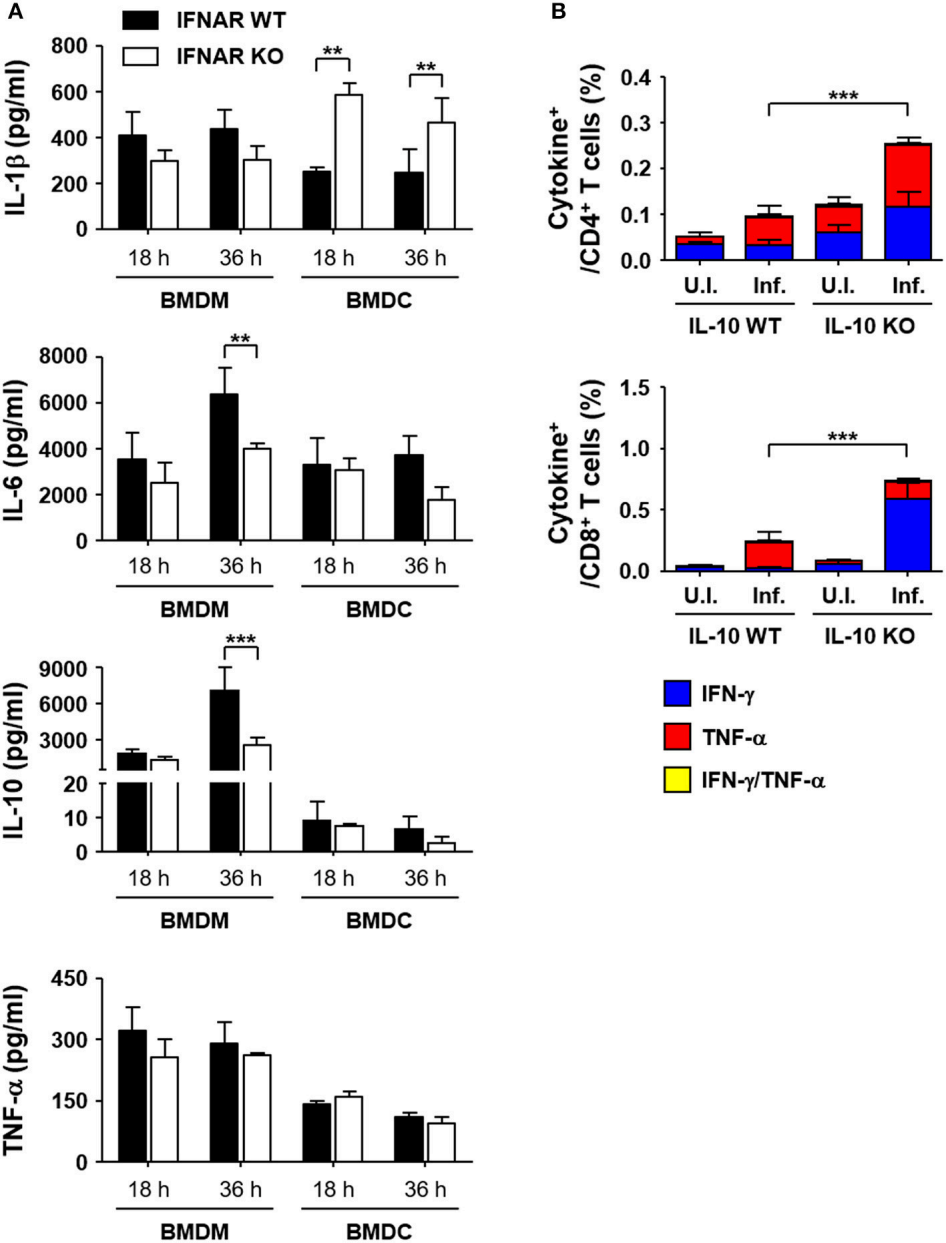
Enhanced expression of IL-10 in macrophages infected with *O. tsutsugamushi* in the absence of type I IFN signaling and its role in T cell responses. **(A)** Levels of cytokines in culture supernatants of BMDMs or BMDCs infected with *O. tsutsugamushi* at the indicated times were analyzed. Phagocytic cells were differentiated from bone marrow cells from wild type (black box) and IFNAR KO (white box) mice and infected with the bacteria (~4 bacteria/cell). Data represent mean + SD from three independent experiments. ^**^p < 0.01; ^***^p < 0.001. (**B**) Splenocytes were collected from wild type and IL-10 KO mice infected with *O. tsutsugamushi*, and analyzed for production of IFN-γ and/or TNF-α in CD4 (upper) or CD8 T (lower) cells by flow cytometry. Splenocytes (2 × 10^6^ cells/24-well) isolated from IL-10 KO mice were infected with *O. tsutsugamushi* for 1 day and further incubated in the presence of tetracycline (0.3 μg/ml) for 3 more days. Then, they were stained with specific antibodies and assessed by flow cytometry. Data represent mean + SD from three independent experiments. ^***^p < 0.001 for IFN-γ-positive T cells. Blue box, IFN-γ-positive; red box, TNF-α-positive; yellow box, IFN-γ and TNF-α-positive. U.I., uninfected; Inf., infected.

To reveal the underlying mechanisms involved in the suppression of T cell responses by type I IFN responses during the acute phase of bacterial infection, we performed *in vitro* infection assays using antigen-presenting phagocytes, BMDMs and BMDCs, from wild type and IFNAR KO mice and measured the levels of inflammatory cytokines produced upon *O. tsutsugamushi* infection (Figure 6A). Among the cytokines expressed by BMDMs infected with *O. tsutsugamushi*, the levels of IL-6 and IL-10 were significantly reduced in cells lacking IFNAR at 36 h after infection when compared to those of wild type macrophages. The production levels of IL-1β and TNF-α were similar in both primary phagocytic cells. In contrast, we observed significantly higher expression levels of IL-1β by IFNAR KO BMDCs than wild type dendritic cells when infected with the bacterial pathogen, whereas secretion of IL-6, IL-10, and TNF-α were similar in both dendritic cell groups. Expression of IL-12 (p70) was barely detectable in phagocytic cells infected with *O. tsutsugamushi* (data not shown). To further delineate the potential roles of the cytokines differentially expressed during the priming of naïve T cells, splenocytes prepared from IFNAR KO mice were infected with *O. tsutsugamushi* for 1 day, treated with tetracycline from the second day, and further incubated for 2 more days. Then, we assessed the percentile of IFN-γ or TNF-α producing CD4 or CD8 T cells by flow cytometry at 4 days after initial infection. In order to assess the potential effect of the inflammatory cytokines during *in vitro* T cell priming, we added specific monoclonal antibodies that neutralize the cytokines to the infection culture (Figure S4 in Supplementary data). Addition of isotype control antibody, anti-IL-1β, IL-6, or IL-10 antibodies did not significantly affect the generation of T cells expressing IFN-γ or TNF-α during *in vitro* infection and T cell priming. However, anti-IL-10 antibody slightly, but not significantly, enhanced the IFN-γ producing CD4 and CD8 T cell fraction when compared to other groups, suggesting that neutralization of IL-10 produced by *O. tsutsugamushi* infection may further increase the generation of antigen-specific T cells during the priming stage in the absence of type I IFN signaling. To further confirm the role of secreted IL-10 in T cell priming, we isolated splenocytes from IL-10 KO mice, infected them with *O. tsutsugamushi*, and analyzed the number of IFN-γ or TNF-α producing T cells as described above. Both CD4 and CD8 T cell populations secreting TNF-α were significantly increased when wild type splenocytes were infected with *O. tsutsugamushi* (*p* < 0.01, Figure 6B). The proportion of CD4 T cells secreting TNF-α or IFN-γ and CD8 T cells producing IFN-γ were further elevated in infected IL-10 KO splenocytes (*p* < 0.001), indicating that expression of IL-10 during naive T cell priming upon infection suppresses the generation of T cell responses.

Taken together, macrophages infected with *O. tsutsugamushi* are the primary source of IL-10, which suppresses the generation of antigen-specific T cells, especially Th1 and CTLs, during the acute phase of infection. Type I IFN responses generated by *O. tsutsugamushi* infection enhances IL-10 secretion from macrophages, thereby impairing the initial priming of T cell responses as well as their long-term memory.

## Discussion

It was previously reported that induction of type I IFNs in monocyte/macrophages by *O. tsutsugamushi* is completely abrogated by heat-inactivation of the pathogen (22, 24). Considering that only live *O. tsutsugamushi* escapes into the cytosol from vesicular compartments or autophagosomes of non-phagocytic and phagocytic host cells within a few hours after infection (31, 37, 38), active evasion from the vesicular compartments of host cells and subsequent exposure to intracellular PRRs is likely a critical opportunity for inducing type I IFNs. Our current results indicate that the RIG-I/MAVS and STING pathways are required for the induction of type I IFNs, but signaling adaptors for TLR pathways, MyD88 and TRIF, are dispensable in phagocytic cells (Figure 2). Since the cytosolic innate sensor systems detect nucleic acids from invading pathogen, active evasion from host trafficking vesicles and sequential release of nucleic acids from the bacteria may be the stimuli of cytosolic intracellular sensors. The ligand is possibly bacterial RNA, DNA, and/or cyclic dinucleotides (3). We also confirmed the role of cyclic GMP-AMP (cGAMP) synthase (cGAS), which detects cytosolic DNA and synthesizes cGAMP to stimulate STING, in expression of type I IFNs by knocking down cGAS in MEFs (Figure S5 in Supplementary data). The involvement of the RIG-I/MAVS and cGAS/STING pathways in the induction of type I IFNs has been well-characterized in another intracellular pathogen, *Listeria monocytogenes*, that replicates in the cytosol of host cell (39, 40). Live *L. monocytogenes* actively escapes from phagosomes by expressing a cytolysin, listeolysin O (LLO), which disrupts the vacuolar membrane (41), and LLO-deficient *L. monocytogenes* fails to induce type I IFNs (39). Bacterial RNA and/or DNA can be released into cytosol either by an active secretion system or by bacteriolysis, which is recognized by RIG-I or IFI16/cGAS sensors, respectively (39, 40). IFI16 and cGAS co-localize with the bacterial DNA in the cytoplasm and are selectively recruited to DNA-activated STING signalosomes (40). Additionally, the downstream signaling components in RIG-I/MAVS and cGAS/STING pathways are physically and functionally interconnected, although distinct classes of receptors are responsible for RNA and DNA sensing (7). For example, the absence of STING expression is renders RIG-I unable to induce type I IFNs in response to cytoplasmic dsRNA (42) and Japanese encephalitis virus infection (43). Involvement of STING in transmitting RIG-I signaling might be mediated by the formation of the RIG-I/MAVS/STING complex upon intracellular pathogen infection (7). Conversely, knockdown of MAVS in host cells markedly reduces phosphorylation of TBK-1 and type I IFNs induced by cytoplasmic DNA (44). These may explain why the absence of one of the signaling components in either pathway abrogates the expression of type I IFNs by *O. tsutsugamushi* infection (Figure 2).

Second question is how nucleic acids of *O. tsutsugamushi* are released from the pathogen. It is interesting to note that mRNA expression of IFN-β peaked at 4 h after infection and decreased thereafter in both MEFs and BMDMs (Figure 1), suggesting a transient activation of type I IFNs during the early stage of intracellular invasion. Transient induction of IFN-β transcripts, peaking at 6 h after infection, in J774A.1 macrophages infected with *O. tsutsugamushi* was also observed in a previous study (22). Considering that escape of the intracellular pathogen from endocytic vesicles into the cytosol generally occurs within 2 h after infection (37), type I IFN transcripts might be transiently induced by *O. tsutsugamushi* during cytoplasmic invasion, but marginally generated during replication in the cytoplasm. Stimulation of nucleic acids-sensing pathways might be reduced during the subsequent cytoplasmic replication stage either by intermittent release of nucleic acids from the cytoplasmic bacteria or by active suppression of the signaling pathways. The true mechanisms induced by *O. tsutsugamushi* remain to be elucidated.

We also examined the potential role of type I IFN responses in *in vivo* pathogenesis of *O. tsutsugamushi* by infecting IFNAR KO mice (Figure 3 and Figures S2, S3). It is clear that the absence of type I IFN responses did not significantly affect the mortality of C57BL/6 mice (Figure 3A and Figure S2). The bacterial burdens in the lungs and spleens of infected mice were slightly higher in IFNAR KO mice, when compared to those of wild type, but not statistically significant (Figure 3B and Figure S3). In addition, the pathological changes of the lungs and spleens of IFNAR KO mice infected with *O. tsutsugamushi*, as well as weight changes, were not significantly different from those of wild type mice. Previously, it was reported that type I IFN induced by *Rickettsia prowazekii* or *Rickettsia conorii* suppresses the bacterial growth *in vitro* infection models (45, 46). However, type I IFN-mediated inhibition of *O. tsutsugamushi* replication *in vitro* varies but never becomes very pronounced (25). It was also shown that Addition of exogenous IFN-α/β (300–450 IU/ml), did not significantly affect the replication of *O. tsutsugamushi* Karp strain in MEFs derived from BALB/c and C3H mice, and Gilliam strain in C3H cells. A 50% decrease of plaque inhibition of Gilliam in BALB/c cells was achieved only with IFN-α/β concentrations over 300 IU/ml and the sensitivity of the Gilliam strain to IFN-α/β was at least 300-fold less than that of Encephalomyocarditis virus (25). Given that the concentration of IFN-β induced by BMDMs infected with *O. tsutsugamushi* Boryong strain, belonging to the Karp genogroup (47), is ~30 pg/ml (Figure 1C, its concentration in plasma of infected mice was always below detection limit throughout the infection period) and 1 IU/ml of type I IFNs generally equals a few pg/ml range (48), physiological levels of type I IFNs induced by *O. tsutsugamushi* infection may not be sufficient to inhibit its replication *in vitro* and *in vivo*. Indeed, when we assessed replication of *O. tsutsugamushi* in BMBMs, there was no significant difference between wild type and IFNAR KO cells (data not shown). Therefore, endogenous type I IFN responses may have only marginal or no direct effects on the bacterial replication, but cytokine responses may critically indirectly influence inflammatory and/or antigen-specific immune responses.

When we assessed the levels of inflammatory cytokines in the sera of infected mice, the concentrations of IFN-γ, IL-6, IL-10, and TNF-α gradually increased in wild type mice, whereas IL-2, IL-4, and IL-12p70 were barely detectable throughout the infection period (Figure 4A and data not shown). Although the cytokine responses in IFNAR KO mice were similar to wild type, the level of IFN-γ was further elevated upon infection with *O. tsutsugamushi*, suggesting enhanced Th1 responses in the absence of type I IFN signaling. This phenotype was confirmed by a significant increase of T-bet^+^CD44^+^ Th1 cell proportion, as well as IFN-γ-secreting activated CD4 T cells, among CD4 T cell population in IFNAR KO mice; however, the levels of CD4 T cell subsets were similarly decreased in both wild type and the mutant mice, potentially via cellular apoptosis, as we observed in scrub typhus patients previously (Figures 4B,C and Table S2) (20). The initial increase of antigen-specific Th1 responses were correlated with significant enhancement of memory T cells, and functionally associated with improved sterile immunity upon secondary bacterial challenge in IFNAR KO mice (Figure 5). Interestingly, TSA56-specific memory antibody (IgG) levels was not affected by the absence of type I IFN signaling, suggesting a specific role in downregulation of cell-mediated immunity, and no effect in humoral immune responses. Elevation of Th1 responses or systemic IFN-γ expression in the absence of type I IFN signaling have also been reported in mice models infected with *Ehrlichia* (49, 50), *Anaplasma* (51), *Chlamydia* (52), *Brucella* (53), and various other intracellular pathogens (3), but the negative effect of type I IFN responses on IFN-γ expression is either beneficial or detrimental to the host, depending on the pathogen. IFNAR KO mice were more resistant to fatal ehrlichiosis than wild type mice (49, 50), whereas *Ananaplasma phagocytophilum* infection was more highly pathogenic in STAT1 KO mice compared to wild type (51). In the case of primary lethal infection with *O. tsutsugamushi*, the magnitude of Th1 responses enhanced by the absence of type I IFN signaling may not be sufficient to provide protective cellular immunity. It was previously reported that CD8 T cell responses are required to protect against lethal infections with *O. tsutsugamushi*, but they also elicit specific pathologic tissue lesions in the liver and lung (54). Nevertheless, suppression of Th1 responses by type I IFNs during primary infection is clearly associated with reduced memory responses of antigen-specific T cells and may restrict sterile immunity against recurrent bacterial infection (Figure 5).

Finally, we tried to elucidate the underlying basis of how type I IFN responses induced by *O. tsutsugamushi* inhibit T cell responses. Among the various potential mechanisms exerted by type I IFNs, elevated IL-10 expression mediated by type I IFN signaling in infected macrophages might be one of the key factors in suppressing T cell responses (Figure 6) (2). The suppressive role of type I IFN/IL-10 during the generation of adaptive T cell immunity has been well-established in various infections (2, 55, 56). For example, IFNAR KO mice are less susceptible to *L. monocytogenes* infection since the intracellular pathogen induces massive apoptotic cell death of lymphocytes by type I IFN sensitization, subsequently leading to IL-10-mediated immunosuppression (55), which can reduce effector Th1 and/or CTL responses and the generation of memory CD4 and CD8 T cells (56, 57). Consistently, IL-10 KO mice are more resistant to *L. monocytogenes* infection, with enhanced control of bacterial replication in spleens and livers (55). In this study, we found that macrophages infected with *O. tsutsugamushi* massively produce IL-10 and wild type BMDMs (~7.0 ng/ml) secrete 2.7 times more IL-10 compared to that of mutant BMDMs lacking IFNAR (~2.6 ng/ml) at 36 h after infection, whereas the levels of IL-10 from BMDCs were generally <10 pg/ml in wild type and IFNAR KO cells (Figure 6A). Although the mechanisms by which type I IFNs promote IL-10 expression in macrophages are not fully understood, a recent study revealed central role of autocrine type I IFNs in increased production of IL-10 and increased IL-10 mRNA stability in macrophages, which is accompanied by increased STAT1 and IRF3 activation (58). The enhanced and prolonged expression of IL-10 is dependent on type I IFN-induced late ERK1/2 phosphorylation (58). Consistent with this, a previous study reported biphasic activation (an initial peak within 10–30 min and persistent activation from 2 to 8 h after infection) of ERK1/2 in murine macrophages supports positive feedback expression of IL-10 by induced type I IFNs (22). We also observed reduced IL-6 expression in IFNAR KO BMDMs at 36 h after infection and enhanced IL-1β secretion in BMDCs lacking IFNAR (Figure 6A). Even though the specific roles and regulatory mechanisms of reduced IL-6 expression in macrophages and enhanced IL-1β expression in dendritic cells during *O. tsutsugamushi* infection in the absence of type I IFN signaling needs to be further defined (2), our pilot study using specific neutralizing antibodies against the cytokines revealed that neutralization IL-10 enhances IFN-γ expression in T cells, whereas antibodies against IL-1β or IL-6 failed to do so (Figure S4). In addition, splenocytes derived from IL-10 KO mice produced significantly higher levels of IFN-γ when compared to those from wild type mice when infected with *O. tsutsugamushi* (Figure 6B). These clearly confirm the suppressive role of IL-10 in naïve T cell priming during bacterial infection. Enhanced expression of IL-10 by *O. tsutsugamushi* infection is consistent in *in vitro* and *in vivo* experiments, as well as studies in scrub typhus patients (10). Moreover, the potential role of IL-10 in the modulation of inflammatory responses and in wide spread tissue damage has also been documented during *O. tsutsugamushi* infection (59, 60).

Taken together, type I IFN responses induced by *O. tsutsugamushi* infection via intracellular nucleic acid sensor pathways in infected host cells contribute to enhanced production of IL-10, which in turn down-modulates antigen-specific T cells and their memory responses. We propose that the type I IFNs/IL-10 axis might play a critical role in suppressing the cellular immunity during acute phase infection, as well as the short longevity of T cell responses, often observed in scrub typhus patients.

## Author contributions

C-KM and N-HC designed the experiments; C-KM, H-IK, N-YH, YK, E-KK, NY, YJ, and K-SI performed the experiments; C-KM, J-IY, YJ, K-SI, M-SC, and N-HC analyzed the results; C-KM, YJ, and N-HC wrote the manuscript; All authors read and approved the final manuscript.

### Conflict of interest statement

The authors declare that the research was conducted in the absence of any commercial or financial relationships that could be construed as a potential conflict of interest.
