# Supplementary material for: A Type I Interferon and IL-10 Induced by Orientia tsutsugamushi Infection Suppresses Antigen-Specific T Cells and Their Memory Responses
Source: Front Immunol. 2018 Sep 4;9:2022. doi: 10.3389/fimmu.2018.02022 (PMC6131522; doi:10.3389/fimmu.2018.02022)
Supplement: Supplementary file 1 [file Data_Sheet_1.doc]

***Supplementary Data***

**A type I interferon and IL-10 induced by *Orientia tsutsugamushi* infection suppresses antigen-specific T cells and their memory responses**

**Chan-Ki Min^1,2,†^, Hong-Il Kim^1,2,†^, Na-Young Ha^1,2^, Yuri Kim^1,2^, Eun-Kyung Kwon^1,2^, Nguyen Thi Hai Yen^1,2^, Je-In Youn^2,3^, Yoon Kyung Jeon^4^, Kyung-Soo Inn^5^,**

**Myung-Sik Choi^1^, and Nam-Hyuk Cho^1,2,3,6,*^**

^1^Department of Microbiology and Immunology, Seoul National University College of Medicine, Seoul, Republic of Korea, ^2^Department of Biomedical Sciences, Seoul National University College of Medicine, Seoul, Republic of Korea, ^3^Wide River Institute of Immunology, Seoul National University College of Medicine, Gangwon-do, Republic of Korea, ^4^Department of Pathology, Seoul National University College of Medicine, Seoul, Republic of Korea, ^5^Department of Pharmaceutical Science, College of Pharmacy, Kyung Hee University, Seoul, Republic of Korea, ^6^Institute of Endemic Disease, Seoul National University Medical Research Center and Bundang Hospital, Seoul, Republic of Korea

^†^ These authors contributed equally.

* Correspondence:

Dr. Nam-Hyuk Cho

chonh@snu.ac.kr

Table S1. The frequencies and absolute counts of T cells in spleens of recovered mice at 6 weeks after infection.


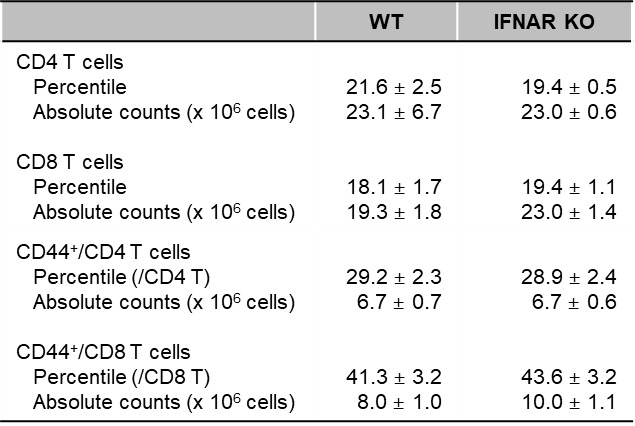


Table S2. The frequencies and absolute counts of total and apoptotic T cells in spleens of mice


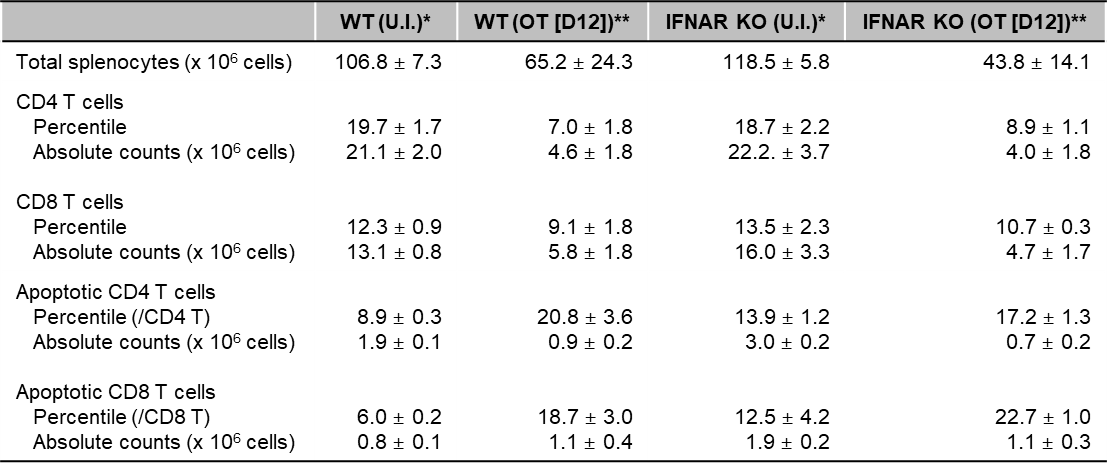


*, U.I., uninfected

**, OT [D12], at day 12 after infection with *O. tsutsugamushi*.


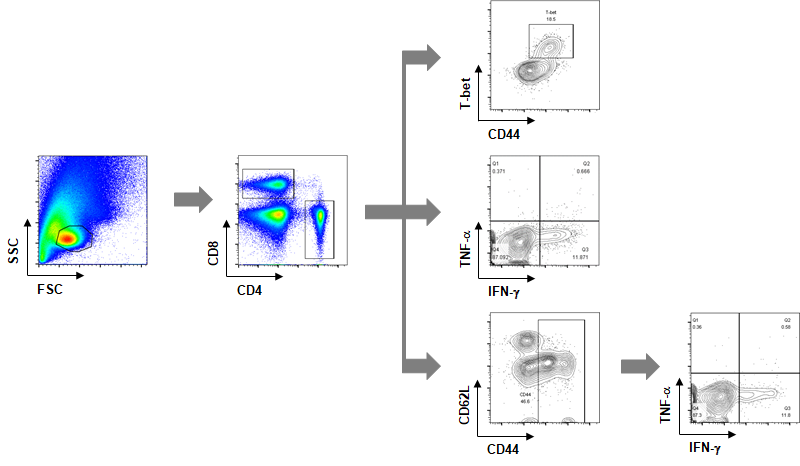


**Figure S1.** Gating strategies for T cell analysis by flow cytometry.


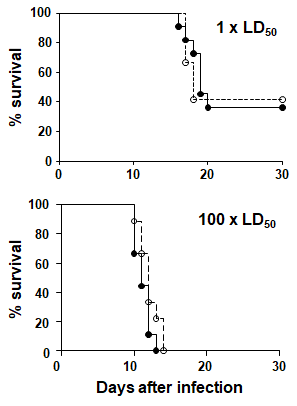


**Figure S2.** Survival rate of wild type (closed circles) and IFNAR KO (open circles) mice infected with different doses of *O. tsutsugamushi* (upper, 1 x LD_50_; lower, 100 x LD_50_). *n* = 10 mice/group.


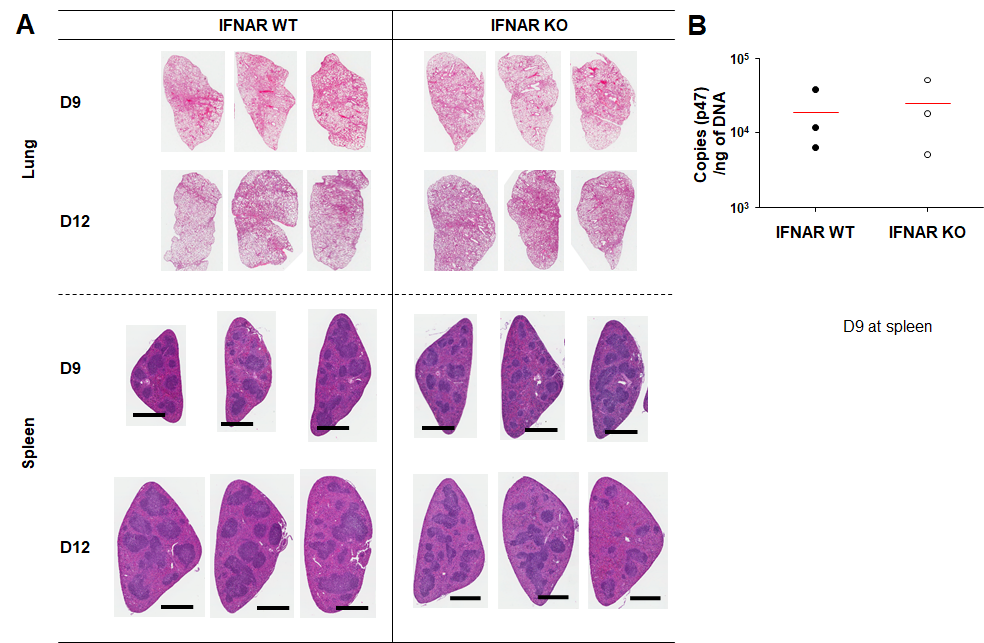


**Figure S3.** Histological pathology of lungs and spleens of infected mice and the bacterial loads in spleens. (**A**) Lung and spleen sections collected from mice at the indicated days after infection were stained with hematoxylin and eosin and representative scanned images are presented. Bar, 1 mm. (**B**) Bacterial loads in the spleens of infected mice (wild type or IFNAR KO, *n* = 3/group) collected 9 days after infection were assessed by qRT-PCR using primer sets detecting the p47 gene of *O. tsutsugamushi*.


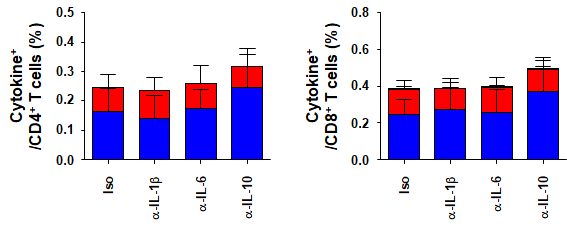


**Figure S4.** Splenocytes were collected from IFNAR KO mice, infected with *O. tsutsugamushi*, and analyzed for production of IFN-γ and/or TNF-α in CD4 or CD8 T cells by flow cytometry. Splenocytes (2 × 10^6^ cells/24-well) isolated from IFNAR KO mice were infected with *O. tsutsugamushi* for one day and further incubated in the presence of tetracycline (0.3 μg/ml) for three more days. Neutralizing monoclonal antibodies, such as anti-IL-1β, anti-IL-6, and anti-IL-10, as well as isotype control antibody (Iso), were added to the culture media (10 μg/ml each/24-well) of the infection assays. Cells were then stimulated with 10 μg of TSA56 for an additional 18 h and 1 μg of Golgiplug for the final 6 h in humidified CO_2_ atmosphere at 37°C. Harvested splenocytes were stained with specific antibodies and analyzed by flow cytometry. Data represent mean + SD from three independent experiments. Blue box, IFN-γ-positive; red box, TNF-α-positive; yellow box, IFN-γ and TNF-α-positive.


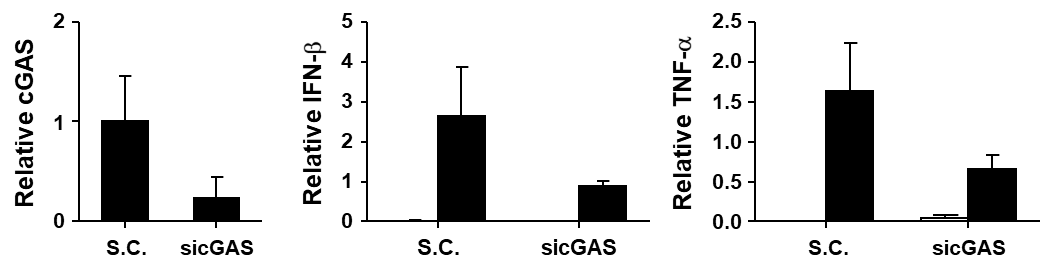


**Figure S5.** Induction of type I IFNs by *O. tsutsugamushi* infection is dependent on cGAS. MEFs were transfected with 20 nM of non-silencing control siRNA (S.C.) or siRNA against cGAS (sicGAS, Sense: CUG AAC ACU GGC AGC UAC U and Antisense: A GUA GCU GCC AGU GUU CAG) using Lipofectamine 2000 (Thermo Fisher Scientific, Waltham, MA). Cells were incubated for 2 more days and infected with *O. tsutsugamushi*. Total RNAs were harvested and relative expressions of cGAS, IFN-β, and TNF-α mRNA were analyzed at 4 h after infection as describe in Fig. 1. Data represent mean + SD from three independent experiments. White box, uninfected; black box, infected.
